# Association of Serum 25-Hydroxyvitamin D Concentration with Breast Cancer Risk in Postmenopausal Women in the US

**DOI:** 10.3390/jpm12060944

**Published:** 2022-06-09

**Authors:** Vijay Ganji, Layan Sukik, Bushra Hoque, Linda Boutefnouchet, Zumin Shi

**Affiliations:** Human Nutrition Department, College of Health Sciences, QU Health, Qatar University, Doha P.O. Box 2713, Qatar; ls1601240@student.qu.edu.qa (L.S.); be1705429@student.qu.edu.qa (B.H.); lb1705990@student.qu.edu.qa (L.B.); zumin@qu.edu.qa (Z.S.)

**Keywords:** breast cancer, cancer, NHANES, National Health and Nutrition Examination Survey, postmenopausal women, serum 25(OH)D, United States, vitamin D

## Abstract

The association between serum 25-hydroxyvitamin D [25(OH)D] concentration and breast cancer risk in postmenopausal women is not well understood. The aim of this study was to investigate the association between serum 25(OH)D concentration and breast cancer in postmenopausal women in the United States using nationally representative sample surveys. We used the data from seven cycles of National Health and Nutrition Examination Surveys from 2001 to 2014. Participants were non-institutionalized postmenopausal women (n = 8108). In restricted cubic spline analysis, a significant, nonlinear, invert ‘U’ relationship was observed between serum 25(OH)D concentrations and breast cancer in postmenopausal women (*p* = 0.029). Overall, breast cancer risk was highest (OR = 1.5) between 70 nmol/L and 80 nmol/L of serum 25(OH)D concentration. Then after serum 25(OH)D 80 nmol/L concentration, the breast cancer risk declined. In multivariate-adjusted logistic regression, the risk of having breast cancer was significantly higher in serum 25(OH)D 75–˂100 nmol/L category compared to the 25(OH)D < 30 nmol/L category [OR and 95% CI: 2.4 (1.4–4.0)]. In conclusion, serum vitamin D concentrations ≥ 100 nmol/L are associated with reduced risk of breast cancer in postmenopausal women. Controlled trials are required to verify if serum 25(OH)D ≥ 100 nmol/L offers protection against breast cancer in postmenopausal women.

## 1. Introduction

Vitamin D is a lipophilic nutrient that is acquired from diet and supplements. Endogenous production of vitamin D occurs in the skin when it is subjected to the sun’s UV (ultraviolet)-B light. The most commonly used biomarker of vitamin D status, as well as the major circulatory form of vitamin D, is 25-hydroxyvitamin D [25(OH)D]. 25(OH)D is hydroxylated to 1,25-dihydroxyvitamin D [1,25(OH)_2_D], a biologically active form, by 1-α hydroxylase in the kidney. All functions of vitamin D are attributed to the action of 1,25(OH)_2_D via vitamin D receptors (VDR). The well-established role of vitamin D is to maintain calcium and phosphorous homeostasis. Recently, the role of vitamin D in non-calcemic functions has received much attention. Many studies have linked serum 25(OH)D concentrations to several chronic diseases such as diabetes [1], obesity [2], metabolic syndrome [1], depression [3], cancer [4], and infectious diseases [5,6].

In the United States (US), the prevalence of vitamin D concentrations such as <30 nmol/L of 25(OH)D, <50 nmol/L of 25(OH)D, and <75 nmol/L of 25(OH)D were 10%, 32% and 76%, respectively [7]. This prevalence of low 25(OH)D concentrations in the US could be due to the increased prevalence of overweight and obesity and the use of sunblock lotions along with decreased milk consumption [8]. Furthermore, previous studies have shown a high prevalence of vitamin D deficiency among postmenopausal women in Western and Asian countries [9,10,11]. In the US, the prevalence of low serum 25(OH)D concentrations (<50 nmol/L of 25(OH)D) among postmenopausal women was 53% [12]. This may be due to decreased estrogen in postmenopausal women [13].

Cancer was found to be one of the leading causes of death worldwide, second only to cardiovascular diseases. Globally, about 9.6 million cancer deaths were reported in 2018 [14]. It has been estimated that in 2020, approximately 1.8 million cancer cases will have been diagnosed in the US population, with around 4950 new cases being diagnosed each day. Among all cancers, breast cancer is the second leading cause of death among women [15]. Several studies have linked vitamin D deficiency with cancer [16,17,18]. A meta-analysis study by Song et al. [16] showed an inverse relationship between serum 25(OH)D concentrations and breast cancer risk in postmenopausal women. They reported that a 5 nmol/L increase in circulating 25(OH)D was associated with a 6% reduction in breast cancer risk. Several other case–control studies conducted on Mexican women and postmenopausal women in Germany indicated a significant inverse relationship between serum 25(OH)D concentrations and breast cancer risk [4,19]. In contrast, a few studies showed null results [20,21].

Overall, the inverse relationship between serum 25(OH)D and cancer is strongly suggestive based on the published reports. It is not known if suboptimal serum 25(OH)D concentrations are related to breast cancer risk in postmenopausal women in the US. Therefore, the aim of this study was to investigate the association between serum 25(OH)D concentration and breast cancer risk in postmenopausal women using the data from the National Health and Nutrition Examination Surveys (NHANES). We hypothesize that serum 25(OH)D would be inversely related to the risk of breast cancer prevalence in postmenopausal women.

## 2. Methods

### 2.1. Description of NHANES

The data for this study are acquired from the multiple 2-year NHANES cycles. NHANES is a periodic study that is conducted by the National Center for Health Statistics (NCHS) on the non-institutionalized civilian population of the US. These are designed to be complex, stratified, multistage, probability sampling surveys to gather data on a sample that represents the US population. People aged ≥60 years old, children, adolescents, and minority groups such as non-Hispanic blacks (NHB) and Mexican American/Hispanic (MA/H) were over-sampled to have more reliable data on these populations. Data on demographic characteristics, health status, and dietary intake were collected using face-to-face interviews, nurse-administered questionnaires, and physical examination. Interviews were conducted in each participant’s house, while the health-related measurements were taken in mobile examination centers (MEC). In MEC, samples of blood and urine were collected to analyze several biochemical markers. The detailed description of methods used can be found elsewhere [22].

### 2.2. NHANES Study Design and Subject Selection

For this cross-sectional study, we used data from 7 cycles of NHANES, 2001–2002, 2003–2004, 2005–2006, 2007–2008, 2009–2010, 2011–2012, and 2013–2014 with reported serum 25(OH)D concentrations. The combined NHANES 2001–2014 yielded 71,037 subjects. Every participant provided “informed consent” prior to taking part in the survey. The NCHS Ethics Review Board approved all survey protocols and consent forms. All procedures were followed in accordance with the relevant guidelines and regulations. The approval number for the NHANESs 2001–2002 and 2003–2004 was #98-12, for the NHANES 2005–2006, 2007–2008, and 2009–2010 was #2005-06, and for the NHANES 2011–2012 and 2013–2014 was #2011-17 [23].

**Figure 1:**
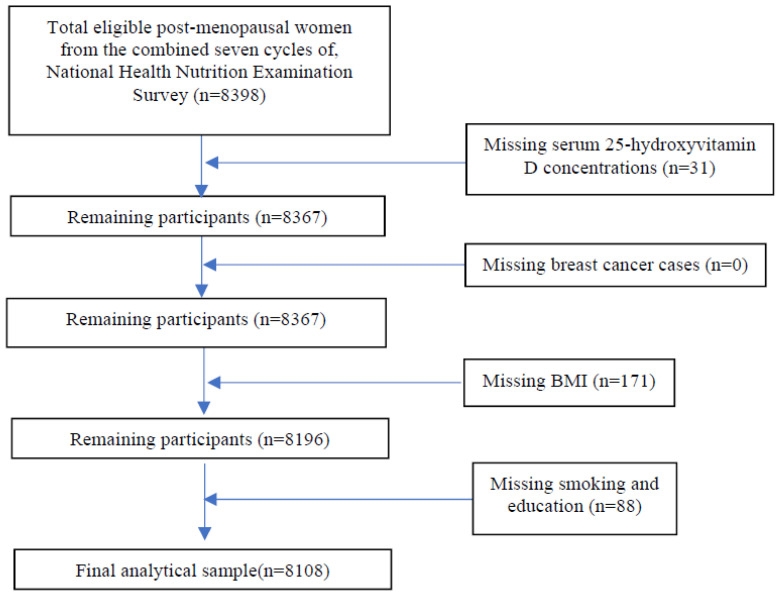
STROBE flow chart: Study sample derivation for the association between serum 25-hydroxyvitamin D concentrations and breast cancer prevalence in postmenopausal women in the USA using the combined data from seven cycles of National Health and Nutrition Examination Surveys, 2001–2014.

Data from the postmenopausal women from combined NHANES 2001–2002 to NHANES 2013–2014 were included in this study. Women who reported that they had the last period 12 months ago or before were considered as women in the postmenopausal state. This method of determination of postmenopausal status is widely used in epidemiological studies [24]. Based on the responses, the total number of women who were considered in a postmenopausal state in 7 cycles of NHANES 2001–2014 was 8398. Subjects having missing data for serum 25(OH)D concentrations (n = 31), breast cancer status (n = 0), or any of the confounding variables (n = 259) were excluded from the study (Figure 1).

### 2.3. Measurement of Serum 25(OH)D Concentrations

We used serum 25(OH)D as a marker of vitamin D status because this represents dietary vitamin D and endogenously synthesized vitamin D in skin. Additionally, it has higher half-life compared to serum 1,25(OH)_2_D. Blood collection by venipuncture was performed according to the standard procedures that were explained in detail in the NHANES Laboratory Procedure Manuals [25,26,27,28]. The measurement of serum 25(OH)D concentration was performed at the NCHS, Centers for Disease Control and Prevention, Atlanta, GA. Laboratory methods for serum 25(OH)D concentration measurement changed from NHANES 2001–2002 to 2013–2014. For NHANES 2001–2002, 2003–2004, and 2005–2006, serum 25(OH)D concentrations were measured with the DiaSorin Radioimmunoassay method (Stillwater, MN, USA) [29]. For NHANES 2007–2008 and 2009–2010, the liquid chromatography-tandem mass spectrometry (LC-TMS) method was employed [25,26]. For NHANES 2011–2012 and 2013–2014, the ultra-high performance LC-TMS method was used [27,28]. In order to make the data compatible across the survey cycles, NCHS used regression modeling to standardize the serum 25(OH)D concentrations.

### 2.4. Outcome Measure: Breast Cancer

Cancer status was assessed using a health questionnaire. Individuals who responded ‘yes’ to the question “Have you ever been told by a doctor or other health professional that you had cancer or a malignancy of any kind?” were considered as a subject having cancer. Further, individuals who responded, “breast cancer” to the question “What kind of cancer was it?” were considered as a subject having breast cancer. Data on specific types of breast cancer were not collected [30].

### 2.5. Confounding Variables

Confounding variables included in this study were age, ratio of family income to poverty [poverty income ratio (PIR)], self-reported race-ethnicity, education level, alcohol consumption, physical activity, smoking status, use of supplements, season of the survey, BMI, and hormone replacement therapy (HRT). BMI and age were used as continuous variables. Remaining variables were used as categorical variables.

### 2.6. Statistical Analyses

The data were analyzed using Stata statistical software package (Version 16, Stata Corporation, College Station, TX, USA) taking sample weights for cumulative 7 cycles of NHANES, 2001–2014 into consideration according to the NCHS analytical guidelines. Serum 25(OH)D concentrations were stratified into 5 categories based on the recommendations by the Institute of Medicine (IOM) [31] and the Endocrine Society (ES) [32]. These cut-off values were <30 nmol/L, 30–<50 nmol/L, 50–<75 nmol/L, 75–<100, and ≥100 nmol/L. Data for subject’s characteristics were presented as n (%) for categorical measures and mean ± SD for continuous measures. The differences between serum 25(OH)D concentration categories were analyzed using chi-squared test for categorical variables and ANOVA for continuous variables.

Unadjusted, age-adjusted, race-ethnicity-adjusted, and multivariate-adjusted logistic regression analyses were performed to determine the association between serum 25(OH)D concentrations and breast cancer prevalence. Confounding variables such as age, education level, HRT, physical activity, the season of survey, smoking status, alcohol consumption, PIR, and BMI were used in the multivariate logistic regression. Additionally, we also evaluated the interaction effects of supplement use (yes and no), season of survey (winter and summer), and race-ethnicity [non-Hispanic white (NHW), NHB, MA/H, and Others] in the association of serum 25(OH)D concentrations with breast cancer in postmenopausal women. Further, we tested the nonlinear relationship between serum 25(OH)D concentrations (used as a continuous variable) and breast cancer using a restrict cubic spline method with three knots at 5th, 50th, and 95th percentiles. Statistical significance was set at a *p*-value of <0.05.

## 3. Results

### 3.1. Sample Derivation and Subject Characteristics

The final analytic sample was 8108 after excluding missing data for 25(OH)D and confounding variables (Figure 1). The characteristics of study participants are presented in Table 1. Age, race-ethnicity, education, smoking status, alcohol intake, season of survey, BMI, supplement intake, HRT, physical activity, and PIR were significantly related to the serum 25(OH)D (*p* < 0.001). In this study, ≈8.6% had serum 25(OH)D < 30 nmol/L, ≈32% had <50 nmol/L, ≈67% had <75 nmol/L, ≈89% had 75–˂100 nmol/L, and 11% had ≥100 nmol/L. In the ≥100 nmol/L serum 25(OH)D concentration category, there were more NHW than NHB or MA/H. Prevalence of <30 nmol/L serum 25(OH)D was the highest in NHB (47%) compared to NHW (≈27%) or MA/H (≈16%). The highest percentage of subjects who participated in the survey in summer was in the serum 25(OH)D ≥ 100 nmol/L category (≈64%), whereas the highest percentage of subjects who were surveyed in winter was in the serum 25(OH)D < 30 nmol/L category (≈58%). About ≈90% of subjects who reported taking supplements a month before the survey was in the 25(OH)D ≥ 100 nmol/L concentration category, while the highest proportion of subjects who did not take supplements was in the lowest 25(OH)D category.

### 3.2. Association between Serum 25(OH)D Concentrations and Breast Cancer Prevalence

**Figure 2:**
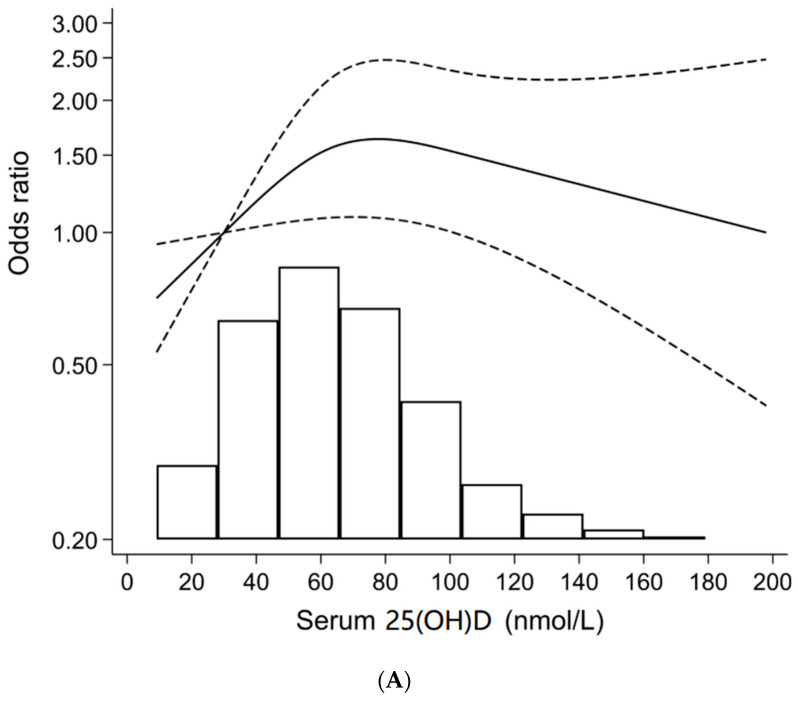

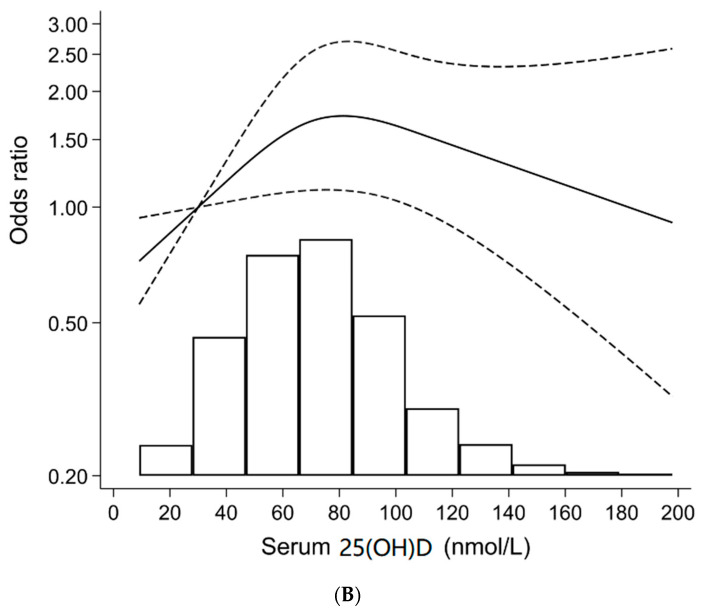
Association between serum 25-hydroxyvitamin D [25(OH)D] concentration and breast cancer prevalence in all postmenopausal women (n = 8108) (**A**) and in non-Hispanic white postmenopausal women (n = 4301) (**B**) using combined National Health and Nutrition Examination Surveys, 2001–2014. Models were adjusted for age, race-ethnicity, poverty income ratio, education, physical activity, season of survey, smoking, alcohol consumption, BMI, and hormone replacement therapy. Restrict cubic spline method was used in the logistic regression to analyze the nonlinear association between serum 25(OH)D concentration and breast cancer prevalence in all women (**A**) (*p* = 0.014) and in non-Hispanic white women (**B**) (*p* = 0.031). Three knots were put at 5th, 50th, and 95th percentiles of serum 25(OH)D concentrations. The risk of breast cancer was highest between 70 nmol/L and 80 nmol/L of serum 25(OH)D concentration in all women (**A**), in non-Hispanic white women (**B**), and in non-Hispanic black women (OR = 1.5). Relation for non-Hispanic blank, Mexican American/Hispanic, and Other Race-Ethnicities is not presented for simplicity.

The nonlinear relationship between breast cancer prevalence and serum 25(OH)D concentrations is presented in Figure 2. Overall, in all women, the nonlinear relationship between serum 25(OH)D concentrations and breast cancer prevalence in the multivariate restrict cubic spline method was significant invert ‘U’ (*p* for nonlinear trend 0.014) (Figure 2A). Further, in the race-ethnicity stratified cubic spline regression analysis, a significant nonlinear association between serum 25(OH)D and breast cancer prevalence was found in NHW postmenopausal women (*p* for nonlinear trend 0.031) (Figure 2B). Overall, the risk of breast cancer was highest (OR ≈ 1.5) between 70 nmol/L and 80 nmol/L of serum 25(OH)D concentration in all women and in NHW. After serum 25(OH)D 80 nmol/L concentration, the risk of breast cancer declined in all women and in NHW. There was no nonlinear relation between breast cancer and serum 25(OH)D in NHB and MA/H (data not shown).

The relationship between serum 25(OH)D concentration (categorized) and breast cancer prevalence in postmenopausal women is shown in Table 2. Overall, in the multivariate-adjusted analysis, the relationship between serum 25(OH)D (categorized) and breast cancer was significant (overall *p* for trend ˂ 0.031). In age-adjusted, race-ethnicity-adjusted, and multivariate-adjusted models, the risk of having breast cancer was highest in the 25(OH)D 75–˂100 nmol/L category. In general, in all three models, the ORs increased from the serum 25(OH)D < 30 nmol/L category to the 25(OH)D 75–˂100 nmol/L category and then decreased in the serum 25(OH)D ≥ 100 nmol/L category. This observed relationship was similar to the invert U relationship that was observed in the cubic spline logistic regression (Figure 2). In the multivariate-adjusted logistic regression analysis, the odds of having breast cancer in the serum 25(OH)D 75–<100 nmol/L category was more than 2 times higher compared to the lowest serum 25(OH)D group (OR = 2.4; 95% 1.4–4.2) (Table 2).

### 3.3. Association between Serum 25(OH)D Concentrations and Breast Cancer Prevalence by Race-Ethnicity, Supplement Use, and Season of Survey

Association between serum 25(OH)D concentration and breast cancer prevalence in postmenopausal women who participated in NHANES 2001–2014 by race-ethnicity is presented in Table 3. Overall, in the multivariate-adjusted logistic regression, there was no association between serum 25(OH)D concentrations and breast cancer in NHW, NHB, and MA/H. Interactions of season of survey, supplement use, and race-ethnicity in the association between serum 25(OH)D and breast cancer in postmenopausal women are presented in Table 4. We found no interaction of supplement use (*p* = 0.95), season of survey (*p* = 0.59), and race-ethnicity (*p* = 0.12) in the relationship between serum 25(OH)D and breast cancer. 

## 4. Discussion

This study is the first to demonstrate a significant nonlinear association between serum 25(OH)D concentrations and breast cancer in postmenopausal women in a nationally representative sample of the US population. The odds of having cancer in postmenopausal women is more than 2 times higher in serum 25(OH)D concentrations of 75–˂100nmol/L compared to the group with ˂30 nmol/L. Moreover, in cubic spline regression analysis, the risk of breast cancer was highest (OR ≈ 1.5) at ≈75 nmol/L of serum 25(OH)D concentrations, with reducing risk after serum 25(OH)D 80 nmol/L. By and large, both analyses are in agreement with the overall invert ‘U’ trend relation between serum 25(OH)D and breast cancer in postmenopausal women.

A limited number of studies have shown a higher risk of breast cancer with increasing serum 25(OH)D concentrations [33,34], although they did not reach statistical significance. A study combining data from three European cohorts of older adults (mean age 63 years) used pre-diagnostic serum 25(OH)D to study the relationship between serum 25(OH)D concentrations and breast cancer risk. These results showed an increase in the risk of breast cancer with 25(OH)D concentrations of >50 nmol/L [33]. Furthermore, a German population-based cohort study reported an increase in breast cancer risk with higher 25(OH)D concentrations during a median 8 years follow-up in women aged 50 to 74 years [34]. Another prospective nested case–control study involving 516 cases and matched controls of postmenopausal women aged 47 to 85 years old from the US found an OR of 1.44 (0.96–2.18) for breast cancer when comparing extreme quartiles of serum 25(OH)D concentrations [35]. On the other hand, several studies reported no relationship between serum 25(OH)D and breast cancer [36,37,38,39,40]. In contrast to our findings, a few studies found an inverse relation between breast cancer prevalence and serum 25(OH)D [4,19,41,42,43]. Conflicting results are more likely due to differences in characteristics of study participants and individual variation in genetics and metabolism.

In the race-ethnicity subgroup analysis, we found a significant nonlinear relation between breast cancer and serum 25(OH)D only in NHW but not in other race-ethnicities. Lack of relation between breast cancer and serum 25(OH)D in NHB and in MA/H was more likely due to lower sample sizes and lower serum 25(OH) concentrations in these race-ethnicities compared to the NHW race [44]. Further studies are warranted in ethnic minorities with a large sample size to confirm these observed associations.

The liver conversion of vitamin D to 25(OH)D is less regulated than the conversion of 25(OH)D to 1,25(OH)_2_D, an active hormone, in the kidney, which suggests that a high serum concentration of 25(OH)D does not necessarily correspond to higher concentrations of 1,25(OH)_2_D [45]. 1,25(OH)_2_D has been shown to contribute to controlling cell growth and proliferation. Vitamin D can be a regulatory factor in the whole process of tumor development starting from early initiation to metastasis. Vitamin D’s anti-inflammatory role and antioxidant defense protects against tumor initiation [46]. It has also been postulated that vitamin D can induce apoptosis in cancer cells, by switching autophagy mode from survival to death mode in these cells [47].

High serum 25(OH)D has been found to be protective against cancer, only when 25(OH)D 1-α hydroxylase (CYP27B1) (converts serum 25(OH)D to 1,25(OH)_2_D) activity is optimum [48]. However, in breast cancer, CYP27B1 expression is reduced, thus limiting the protection that can be afforded by serum 25(OH)D [49,50]. In breast tissues, it has been shown that with tumor progression, there was an increase in 1,25(OH)_2_D 24-hydroxylase (CYP24A1) expression and a decrease in CYP27B1 expression [50]. CYP24A1 enzyme is responsible for catalyzing hydroxylation reactions leading to the degradation of 1,25(OH)_2_D to inactive 24,25(OH)_2_D. The *CYP24A1* gene has been observed to amplify in breast cancer [51]. Additionally, the results of Townsend et al. have shown that compared with normal breast tissue, there was an upregulation of CYP24A1 mRNA in breast tumor tissue [52]. The concentration of 25(OH)D present in tissues depends on the synthesis of CYP27B1 and the degradation of CYP24A1. A decrease in regulation for both enzymes has been seen in multiple stages of breast cancer development [53]. Accordingly, the expression of VDR, CYP27B1, and CYP24A1 in breast cancer may favor tumor progression by decreasing the presence of the active 1,25(OH)_2_D. Nonetheless, it is not known at what concentrations 25(OH)D is protective against breast cancer.

Overall, results from studies reporting the association of serum 25(OH)D with the prevalence/incidence of breast cancer are equivocal. Recent evidence from meta-analysis studies suggested an inverse association of serum 25(OH)D (not dietary vitamin D intake) with breast cancer [16,54]. In a study by Huss et al. [55], pre-diagnostic high serum 25(OH)D was associated with higher risk for breast cancer death than those with intermediate serum 25(OH)D. Vitamin D elicits its biological function through binding to VDRs. Breast cancer cells express more VDRs than normal cells [56]. High VDR expression was associated with improved prognosis and decreased mortality from breast cancer [57]. In addition, it has been found that breast cancer cells overexpress macroH2A, a differentiation promotor. Overexpression of this factor is related to poor prognosis and increased metastasis of breast cancer [58]. It is not known whether high serum 25(OH)D leads to higher expression of VDRs or macroH2A in breast cancer cells.

The findings from this study should be viewed in terms of a few limitations. Due to the cross-sectional nature of this study, the cause-and-effect relation between serum vitamin D and breast cancer should not be assumed. In this study, the majority of the breast cancer diagnosis must have happened before the serum 25(OH)D measurement. There were no data on for how long the women had been diagnosed with breast cancer before the serum 25(OH)D measurement was taken. One cross-sectional measurement of serum 25(OH)D concentration may not reflect the concentrations many years prior especially in those who had a major change in their health status (such as cancer diagnosis). Therefore, it may be that some cancer patients after cancer diagnosis started taking vitamin D supplements or modified their diets to include more vitamin D-rich foods. To what extent this affected the results is not known. Additionally, it is possible that breast cancer therapy may have affected the circulating 25(OH)D concentration. It has been reported that the treatment of early stages of breast cancer using anti-hormone therapy with tamoxifen has been shown to increase serum 25(OH)D [59]. It is not known how many subjects and how long subjects were on tamoxifen in this study population. However, these data are not available in NHNAES. Overall, several study participants reported consuming supplements, with some of them having serum 25(OH)D concentrations ≥ 75 nmol/L. However, the impact of this on the current results is less likely as we did not find the interaction effect of supplement use in the association of serum 25(OH)D with breast cancer prevalence. Because the breast cancer was self-reported by participants, there is a possibility of misclassification of cases. Although race-ethnicity and season of the survey are known to affect the serum 25(OH)D concentrations, in the multivariate regression analysis, we found no interaction of these variables with the association of serum 25(OH)D with breast cancer.

A strength of this study includes a combined seven cycles of NHANES based on a nationally representative sample. This makes the results of this study generalizable to the population described in this study. Current data analysis was based on standardized 25(OH)D concentrations which allowed us to concatenate various cycles of NHNAES into one large database. This increased the precision of the estimate. Additionally, we used clinically meaningful cut-off values based on the ES and the IOM recommendations in assessing the association between serum 25(OH)D and breast cancer rather than artificial cut-off quartiles or tertiles.

## 5. Conclusions

In conclusion, an invert ‘U’ association was observed between serum 25(OH)D concentrations and breast cancer in postmenopausal women. It appears that serum 25(OH)D concentrations ≥ 100 nmol/L may be protective against breast cancer in postmenopausal women. Controlled trials are needed to study the association between serum 25(OH)D and breast cancer risk and to further elucidate the mechanism of vitamin D in cancer pathogenesis in postmenopausal women.

## Figures and Tables

**Table 1:** Sample characteristics of study population by serum 25-hydroxyvitamin D [25(OH)D] concentrations: National Health and Nutrition Examination Surveys 2001–2014 (n = 8108) ^1^.

Serum 25(OH)D Concentrations ^2^						
	<30 nmol/L	30–<50 nmol/L	50–<75 nmol/L	75–<100 nmol/L	≥100 nmol/L	*p*-Value ^3^
	(n = 697)	(n = 1931)	(n = 2778)	(n = 1822)	(n = 880)	
Serum 25(OH)D, nmol/L	23.6 (4.6)	40.4 (5.8)	62.1 (7)	85.8 (7.1)	122.2 (22.8)	<0.001
Age, y	61.4 (12.5)	62.1 (12.9)	63 (12.9)	64.1 (12.6)	65.8 (12.4)	<0.001
Race-ethnicity						<0.001
Non-Hispanic white, n (%)	189 (27.1)	744 (38.5)	1496 (53.9)	1223 (67.1)	649 (73.8)	
Non-Hispanic black, n (%)	327 (46.9)	553 (28.6)	417 (15)	204 (11.2)	98 (11.1)	
Mexican American/Hispanic, n (%)	114 (16.4)	389 (20.1)	459 (16.5)	159 (8.7)	30 (3.4)	
Others, n (%) ^4^	67 (9.6)	245 (12.7)	406 (14.6)	236 (13)	103 (11.7)	
Smoking status						<0.001
Never, n (%)	329 (47.2)	1148 (59.5)	1690 (60.8)	1080 (59.3)	514 (58.4)	
Former, n (%)	176 (25.3)	449 (23.3)	695 (25)	530 (29.1)	263 (29.9)	
Current, n (%)	192 (27.5)	334 (17.3)	393 (14.1)	212 (11.6)	103 (11.7)	
Alcohol drinking ^5^						<0.001
No, n (%)	216 (31)	524 (27.1)	687 (24.7)	394 (21.6)	191 (21.7)	
Yes, n (%)	312 (44.8)	883 (45.7)	1405 (50.6)	1033 (56.7)	503 (57.2)	
Not reported, n (%)	169 (24.2)	524 (27.1)	686 (24.7)	395 (21.7)	186 (21.1)	
Season of survey						<0.001
Winter, n (%)	406 (58.2)	1027 (53.2)	1219 (43.9)	691 (37.9)	321 (36.5)	
Summer, n (%)	291 (41.8)	904 (46.8)	1559 (56.1)	1131 (62.1)	559 (63.5)	
Body mass index, kg/m^2^	32.6 (8.3)	31.2 (7.2)	29.4 (6.7)	28.4 (6.8)	27.3 (5.9)	<0.001
Physical activity (METs/week) ^6^						<0.001
<600 (low), n (%)	488 (70)	1210 (62.7)	1512 (54.4)	891 (48.9)	428 (48.6)	
600–1199 (moderate), n (%)	65 (9.3)	229 (11.9)	385 (13.9)	256 (14.1)	124 (14.1)	
≥1200 (vigorous), n (%)	144 (20.7)	492 (25.5)	881 (31.7)	675 (37)	328 (37.3)	
Poverty Income Ratio ^7^						<0.001
<1.30 (low income), n (%)	254 (36.4)	621 (32.2)	799 (28.8)	429 (23.5)	169 (19.2)	
1.3–3.5 (moderate income), n (%)	245 (35.2)	728 (37.7)	1034 (37.2)	607 (33.3)	326 (37)	
>3.5 (high income), n (%)	132 (18.9)	420 (21.8)	730 (26.3)	644 (35.3)	324 (36.8)	
Not reported, n (%)	66 (9.5)	162 (8.4)	215 (7.7)	142 (7.8)	61 (6.9)	
Education						<0.001
<11 grade, n (%)	274 (39.3)	681 (35.3)	891 (32.1)	399 (21.9)	156 (17.7)	
High school, n (%)	165 (23.7)	482 (25)	682 (24.6)	509 (27.9)	229 (26)	
Some college, n (%)	179 (25.7)	521 (27)	748 (26.9)	534 (29.3)	255 (29)	
Higher than College, n (%)	79 (11.3)	247 (12.8)	457 (16.5)	380 (20.9)	240 (27.3)	
Hormone replacement therapy, n (%) ^8^	106 (15.2)	339 (17.6)	582 (21)	291 (16)	63 (7.2)	<0.001
Supplements intake ^9^						<0.001
Yes, n (%)	200 (28.7)	861 (44.5)	1902 (68.5)	1488 (81.7)	790 (89.8)	
No, n (%)	497 (71.3)	1070 (55.5)	875 (31.5)	334 (18.3)	90 (10.2)	

^1^ Weighted n = 296,383,878. National Health and Nutrition Examination Surveys, 2001–2002, 2003–2004, 2005–2006, 2007–2008, 2009–2010, 2011–2012, and 2013–2014 were combined into one analytic database (2001–2014) for this analysis. Data are presented as mean ± SD for continuous measures and n (%) for categorical measures. ^2^ Serum 25(OH)D concentrations <30 nmol/L, 30–<50 nmol/L, ≥50 nmol/L are considered deficient, insufficient, and sufficient according to the Institute of Medicine. Serum 25(OH)D concentrations <50 nmol/L, 50–<75 nmol/L, and ≥75 nmol/L are considered deficient, insufficient, and sufficient according to the Endocrine Society. Conversion, 1 nmol/L = 0.4066 ng/mL. ^3^ Significance in the analysis of variance for continuous measures or χ^2^ test for categorical measures. ^4^ Included non-Hispanic Asians and multi-racial subjects. ^5^ Collected from the responses to the question “Have you had at least 12 alcohol drinks/1 year?”. ^6^ Measured according to the time usually spent sitting during a typical day. This time included sitting at work, home, or school, and time spent sitting with friends, in a car, bus, or train, when reading, watching television, or when using a digital device. Time spent sleeping was not included. ^7^ Calculated by dividing the family’s income over the family’s poverty threshold. ^8^ Data were collected from the questions “have you used female hormones such as estrogen and progesterone for menopause-related symptoms (hot flashes, sweating, vaginal dryness, bladder problems); depression, anxiety, emotional distress; hysterectomy or oophorectomy (ovary removal); osteoporosis, bone loss/thinning fracture prevention; cardiovascular disease prevention; irregular menstrual periods/to regulate periods; and other reasons?” If a participant answered ‘yes’ to at least one of these questions, that participant was considered as using hormone replacement therapy. ^9^ Participants who answered ‘yes’ to the question “Have you used or taken any vitamins, minerals, or other dietary supplements in the past month?” were considered as supplements users.

**Table 2:** Association between serum 25-hydroxyvitamin D [25(OH)D] concentrations and breast cancer prevalence in postmenopausal women in the USA: National Health and Nutrition Examination Surveys 2001–2014 (n = 8108) ^1^.

Serum 25(OH)D Concentrations ^2^						
	<30 nmol/L ^3^ (n = 697)	30–<50 nmol/L (n = 1931)	50–<75 nmol/L (n = 2778)	75–<100 nmol/L (n = 1822)	≥100 nmol/L (n = 880)	*p* for Trend ^4^
Breast cancer cases, n (%)	23 (3.3)	77 (4)	128 (4.6)	117 (6.4)	61 (6.9)	<0.001
Unadjusted	1	1.7 (0.98–3)	2 (1.2–3.4) ^5^	2.7 (1.6–4.5) ^5^	2.4 (1.4–4.2) ^5^	<0.001
Age-adjusted	1	1.7 (0.95–2.9)	1.9 (1.1–3.3) ^5^	2.5 (1.5–4.2) ^5^	2 (1.2–3.6) ^5^	0.005
Race-ethnicity-adjusted	1	1.7 (0.94–2.9)	1.8 (1.1–3.1) ^5^	2.4 (1.4–4) ^5^	2.1 (1.2–3.7) ^5^	0.007
Multivariate-adjusted ^6^	1	1.7 (0.9–3)	1.9 (1.05–3.3) ^5^	2.4 (1.4–4.2) ^5^	1.94 (1.03–3.6) ^5^	0.031

^1^ National Health and Nutrition Examination Surveys, 2001–2002, 2003–2004, 2005–2006, 2007–2008, 2009–2010, 2011–2012, and 2013–2014 were combined into one analytic database (2001–2014) for this analysis. Values are odds ratios and their 95% confidence intervals. ^2^ Serum 25(OH)D concentrations <30 nmol/L, 30–<50 nmol/L, ≥50 nmol/L are considered deficient, insufficient, and sufficient according to the Institute of Medicine. Serum 25(OH)D concentrations <50 nmol/L, 50–<75 nmol/L, and ≥75 nmol/L are considered deficient, insufficient, and sufficient according to the Endocrine Society. Conversion, 1 nmol/L = 0.4066 ng/mL. ^3^ Referent category. ^4^ Significance for the trend in the logistic regression between serum 25(OH)D concentration clinical cut off points and breast cancer prevalence. ^5^ Significantly different from referent (25(OH)D < 30 nmol/L) category. ^6^ Adjusted for age, race-ethnicity, poverty income ratio, education, physical activity, season of survey, smoking, alcohol consumption, BMI, and hormone replacement therapy.

**Table 3:** Association between serum 25-hydroxyvitamin D [25(OH)D] concentrations and breast cancer prevalence by race-ethnicity in the USA: National Health and Nutrition Examination Surveys 2001–2014 (n = 8108) ^1^.

Serum 25(OH) D Concentrations ^2^						
	<30 nmol/L ^3^	30–<50 nmol/L	50–<75 nmol/L	75–<100 nmol/L	≥100 nmol/L	*p* for Trend ^4^
Non-Hispanic white (n = 4301)						
Unadjusted	1	3 (1.1–7.4) ^5^	3 (1.1–7.4) ^5^	3.7 (1.5–9.1) ^5^	3 (1.2–7.8) ^5^	0.049
Age-adjusted	1	3 (1.1–7.5) ^5^	3 (1.2–7.4) ^5^	3.8 (1.6–9.3) ^5^	3 (1.1–7.3) ^5^	0.1
Multivariate-adjusted ^6^	1	3 (1.14–8) ^5^	3.14 (1.2–8.3) ^5^	4.1 (1.6–11) ^5^	3.2 (1.15–8.7) ^5^	0.07
Non-Hispanic black (n = 1599)						
Unadjusted	1	0.6 (0.3–1.3)	0.6 (0.3–1.4)	1.6 (0.6–4.2)	2.2 (0.8–6)	0.11
Age-adjusted	1	0.6 (0.3–1.3)	0.6 (0.2–1.3)	1.3 (0.5–3.5)	1.7 (0.6–4.9)	0.23
Multivariate-adjusted ^6^	1	0.6 (0.3–1.3)	0.56 (0.25–1.3)	1.4 (0.53–3.8)	1.8 (0.6–5.3)	0.25
Mexican American/Hispanic (n = 1151)						
Unadjusted	1	3 (0.5–18)	2.6 (0.3–22)	0.8 (0.1–8.2)	2.2 (0.1–35)	0.56
Age-adjusted	1	3 (0.5–18)	2.6 (0.3–22)	0.7 (0.1–8.1)	2.1 (0.1–36)	0.53
Multivariate-adjusted ^6^	1	2 (0.33–12)	2 (0.3–12)	0.5 (0.04–6)	1.2 (0.1–25)	0.35

^1^ National Health and Nutrition Examination Surveys, 2001–2002, 2003–2004, 2005–2006, 2007–2008, 2009–2010, 2011–2012, and 2013–2014 were combined into one analytic database (2001–2014) for this analysis. Values are odds ratios and their 95% confidence intervals. ^2^ Serum 25(OH)D concentrations <30 nmol/L, 30–<50 nmol/L, ≥50 nmol/L are considered deficient, insufficient, and sufficient according to the Institute of Medicine. Serum 25(OH)D concentrations < 50 nmol/L, 50–<75 nmol/L, and ≥75 nmol/L are considered deficient, insufficient, and sufficient according to the Endocrine Society. Conversion, 1 nmol/L = 0.4066 ng/mL. ^3^ Referent category. ^4^ Significance in the logistic regression between serum 25(OH)D concentration clinical cut off points and breast cancer prevalence. ^5^ Significantly different from referent (25(OH)D < 30 nmol/L) category. ^6^ Adjusted for age, poverty income ratio, education, physical activity, season of survey, smoking, alcohol consumption, BMI, and hormone replacement therapy.

**Table 4:** Association of breast cancer with serum 25-hydroxyvitamin D [25(OH)D] concentrations according to supplement use, season of survey, and race-ethnicity in postmenopausal women in the USA: National Health and Nutrition Examination Surveys 2001–2014 (n = 8108) ^1^.

Demographic Characteristic	Serum 25 (OH)D Concentrations ^2^					
	<30 nmol/L ^3^	30–˂50 nmol/L	50–˂75 nmol/L	75–˂100 nmol/L	≥100 nmol/L	*p* for Interaction ^4^
	OR	OR (95%CI)	OR (95%CI)	OR (95%CI)	OR (95%CI)	
Supplement use						0.95
Yes	1	2.1 (0.7–6)	2.3 (0.8–7)	3 (1.1–8)	2.5 (0.8–7)	
No	1	1.4 (0.7–3)	1.5 (0.6–3.6)	2.1 (0.99–5)	1.3 (0.4–4.4)	
Season of survey						0.59
Winter	1	2.3 (0.9–8-5)	2.7 (1.1–7)	3.1 (1.4–7)	2.4 (0.85–7)	
Summer	1	1.3 (0.6–3)	1.3 (0.6–3)	1.8 (0.8–4)	1.4 (0.6–3.3)	
Race-ethnicity						0.12
Non-Hispanic white	1	3 (1.1–8)	2.9 (1.1–8)	3.7 (1.4–10)	2.9 (0.99–8)	
Non-Hispanic black	1	0.6 (0.3–1.4)	0.6 (0.3–1.4)	1.5 (0.6–4)	2 (0.6–6)	
Mexican American/Hispanic	1	2 (0.3–11)	1.8 (0.3–10)	0.4 (0.04–5)	1.1 (0.1–21)	
Others	1	1.1 (0.1–13)	3 (0.3–32)	2.6 (0.3–26)	2.8 (0.3–28)	

^1^ National Health and Nutrition Examination Surveys, 2001–2002, 2003–2004, 2005–2006, 2007–2008, 2009–2010, 2011–2012, and 2013–2014 were combined into one analytic database (2001–2014) for this analysis. Values are odds ratios and their 95% confidence intervals. ^2^ Serum 25(OH)D concentrations <30 nmol/L, 30–<50 nmol/L, ≥50 nmol/L are considered deficient, insufficient, and sufficient according to the Institute of Medicine. Serum 25(OH)D concentrations < 50 nmol/L, 50–<75 nmol/L, and ≥75 nmol/L are considered deficient, insufficient, and sufficient according to the Endocrine Society. Conversion, 1 nmol/L = 0.4066 ng/mL. ^3^ Referent category. ^4^ Multivariable adjusted interaction effect in the logistic regression between serum 25(OH)D concentration clinical cut-off points and breast cancer prevalence according to supplement use, season of survey, and race-ethnicity.

## Data Availability

Data used in this study are available from Z.S. upon request.

## Reporting Guidelines

This manuscript was prepared according to STROBE guidelines.

## Abbreviations

[1,25(OH)_2_D]	1,25-dihydroxyvitamin D
CYP24A1	24-hydroxylase
[25(OH)D]	25-hydroxyvitamin D
CYP27B1	25-hydroxyvitamin D 1-α hydroxylase
ES	Endocrine Society
HRT	hormone replacement therapy
IOM	Institute of Medicine
LC-TMS	Liquid Chromatography-Tandem Mass Spectrometry
MET	Metabolic Equivalent of Task
MA/H	Mexican American/Hispanic
MEC	Mobile Examination Centers
NHANES	National Health and Nutrition Examination Survey
NHB	non-Hispanic black
NCHS	National Center for Health Statistics
PIR	poverty income ratio
UV	Ultraviolet
US	United States
VDR	vitamin D receptors
